# Process development for antifungal production by *Bacillus subtilis* BS20: nanoparticle supplementation, process optimization and preliminary scale-up studies

**DOI:** 10.1007/s00449-025-03205-6

**Published:** 2025-07-26

**Authors:** Sikhulile N. Nzimande, Isaac A. Sanusi, Kwasi Yobo, Santosh O. Ramchuran, Gueguim E. B. Kana

**Affiliations:** 1 School of Life Sciences, University of KwaZulu-Natal, Pietermaritzburg, South Africa; 2 School of Agriculture Earth & Environmental Sciences, University of KwaZulu-Natal, Pietermaritzburg, South Africa; 3 School of Life Sciences, University of KwaZulu-Natal, Westville, South Africa

**Keywords:** Antifungal activity, Nanobiocatalyst, Kinetic model, Bioreactor, Impeller tip speed, Scale up

## Abstract

The intensive agricultural practices used to meet global crop production demands have resulted in rigorous use of chemical pesticides. These ultimately compromise crop production as well as the environment. To alleviate these challenges, cheaper and environmentally friendly biocontrol agents have been considered as an alternative to chemical pesticides. Hence, this study was undertaken with the aim of enhancing antifungal production by *Bacillus subtilis* BS20 through process modeling, optimization, nanocatalysis and subsequent assessment of the scale up potential of the optimized process. The investigated process parameters included glucose concentration (10–30 g/L), incubation temperature (25–45 ℃) and incubation time (24–96 h). Optimized process conditions of 11.5 g/L glucose concentration, 24 h incubation time and 41 °C incubation temperature produced maximal antifungal activity of 68 mm zone of inhibition. Moreover, the inclusion of nanoparticles favored increased biomass yield but low antifungal activity. Additionally, constant power consumption, Reynolds number (Re) and impeller tip (*V*_*tip*_) speed were implemented to scale up the antifungal production by *B. subtilis* BS20. Implementing constant *V*_*tip*_ value from the 1 L scale: 93 rpm, Re = 5.9E-04, Power (P) = 0.32 W, Power to Volume ratio (P/V_L_) = 160 W/m^3^, circulation time (*t*_*c*_) = 5.2 s and shear stress (γ) = 15.5 S^−1^, at 41 °C, gave the highest antifungal activity of 65 mm zone of inhibition in the 10 L scale bioreactor compared to the 1L bioreactors (57 mm). These findings have elucidated improved antifungal production by *B. subtilis* BS20 as well as provided a preliminary data for large scale production.

**Supplementary Information:**

The online version contains supplementary material available at 10.1007/s00449-025-03205-6.

## Introduction

The extensive use of agricultural chemicals and pesticides to protect plants from pests, weed or diseases have been associated with negative effects on crop productivity, foodborne illness and soil toxicity [4, 30, 42]. The development and implementation of sustainable and ecological approach has become imperative to alleviate the risk that chemical pesticides pose to humans and the environment. Eco-friendly alternatives such as biological control agents have gained considerable attention, the implementation of beneficial microorganisms such as *Bacillus* spp. in this regard is considered one of the most promising methods [37]. Eco-friendly practices are crucial for the attainment of sustainable commercial scale crop production. Additionally, there is global increase in food demand due to overall growth in world population. This has made the development of a biobased food loss control such as use of antifungal metabolite an imperative approach in mitigating the negative impact of synthetic chemicals.

Antifungal metabolites are one of the most important bioproducts used in various industries such as agricultural industry [17, 18]. They are natural biomolecules produced by microorganism such as *Bacillus* spp. which have gained interest from various industries for their eco-friendliness. These antifungal metabolites with surfactant properties consists of hydrophilic and lipophilic moieties. These compositions give rise to their biological activities such as antibacterial, antifungal, antiviral, and cytolytic activities [18]. These have become attractive for biological control of microbial food spoilage since chemical pesticides are toxic and non-biodegradable [10, 23]. Also, the global issue of microbial food spoilage has resulted in major effects such as crop spoilage and foodborne illnesses [5]. Thus, biological based food pathogens (like *Rhizoctonia solani*) control such as the use of surface active lipopeptides has become indispensable. *R. solani* possesses a wide host range and can cause diseases in many crop plants that can result to huge economic losses [12]. A major challenge with *R. solani* related diseases is that it is difficult to manage because of its soil- and seed-borne potentials as well as the easy of their being borne by plant organs [6, 7]. Hence, the main difficulties faced in the control of *Rhizoctonia* pathogen are its large host range. This pathogen causes huge yield losses by affecting seed, root, stem, leaf, and fruit (common examples are fruit rot, stem and crown cancer, leaf and scabbard blight, as well as dwarfism in plant) [6]. Due to this devastating potential of *Rhizoctonia* pathogen a lot of chemical treatment has been applied to control its impact. This approach is not environmentally friendly hence biological strategies such as in the present study is desirable from both environmental, food quality and economic point of view. Biological control is one of the important alternative strategies being implemented to combat the menace of *R. solani* diseases. This biological control using microbial products or metabolites directly interact with *R. solani* via interactions like hyper parasitism, antibiosis, competition and triggered host defense reaction or mechanism of host plants to control the pathogen. Important fungi species regarding *R. solani* biological control are the general of *Trichoderma*, *Gliocladium*, *Verticillium*, and *Stachybotrys*. While bacteria species implicated in *R. solani* biological control are the general of *Bacillus*, *Streptomyces* and *Pseudomonas* [6]. Nevertheless, there is limit report on the use of antifungal metabolite produced from *B. subtilis* BS20 to combat *R. solani* growth towards combating their destructive impact.

*Bacillus* species are known for their ability to synthesize a variety of antimicrobial compounds that have shown variable antagonistic effects against bacterial and fungal phytopathogens. However, the production processes of these antimicrobial compounds have not reached the desirable yields. Moreover, there is a dearth of study on improving antifungal yield. Presently, *Bacillus* spp. naturally produce antifungal compounds at low quantities which has driven researcher to make notable efforts to enhance their production quantitatively and qualitatively. These antifungal metabolites are influenced by operational parameters such as carbon source, pH, incubation time and temperature [15, 18, 27]. Despite the abundance of previous studies on key input parameters, determining optimum parameter conditions that effectively facilitates microbial growth and metabolism for antifungal production, there is a dearth of report on *Bacillus subtilis* BS20 process modeling, optimization, nano-catalysis and subsequent assessment of the scale up potential for high yield antifungal metabolites. Generally, antifungal yields are affected by process conditions such as culture media, nutrient supplementation, pH and temperature [15]. Only a few studies have reported on the interactive effect and impact of operating conditions on the dynamic behavior of antifungal production [18, 27]. Therefore, the application of optimization tools to ensure an improved overall process performance and high antifungal yield as well as high product quality at lower process cost is desirable.

Aside the use of process optimization tools, biocatalytic materials such as nanoparticles have recently been employed to improve bioprocess productivity. Nanoparticles have gained much attention in biomedical science, environmental science, biotechnology, optics, magnetics, catalysis, and energy science due to their unmatched physical and chemical properties compared to their macro counterparts. The application of nano-biocatalyst agents in bioprocessing is to enhance process productivity through increased mass and heat transfer, enzymatic and cell metabolic activities arising from their large surface areas, catalytic properties [1]. Furthermore, nanoparticles are known to improve the capacity of electron donor reactions towards enhanced biological activity of microbes [2, 39, 43]. Yet, there is scarcity of study on nano-supplementation application in antifungal production due to poor understanding of such processes.

Moreover, the effect of process scale up could significantly impact the process kinetics and ultimately the process productivity efficiency [44]. Currently, there is scarcity of studies on process scaling up and the kinetics of *Bacillus* based antifungal compound production. Although, several biotechnological challenges have been resolved in relatedness to microbial engineering for biobased antimicrobial production, knowledge-based bioreactor design for efficient scale up of *Bacillus* based antifungal production is still underdeveloped.

Therefore, the objectives of this study are (1) use response surface methodology (RSM) to capture the complex interactions which link the process conditions to antifungal production, (2) explore the supplementation of nanobiocatalyst for enhance antifungal production, and (3) examine the various scale up criterion at different scales on antifungal production by *B. subtilis* BS20.

## Materials and methods

### Microorganisms and inoculum preparation

*Bacillus subtilis* BS20 obtained from the Discipline of Microbiology, University of KwaZulu-Natal, Durban, South Africa, was used in this study. A single flask containing 100 mL Luria Bertani (LB) medium was inoculated with a single colony of the respective *B. subtilis* for inoculum development and grown at 120 rpm, 30 °C for 24 h. The culture was then used as the inoculum (10% v/v; ~ 1 × 10^6^ cells/mL) for subsequent fermentation processes in the antifungal production.

### Modeling and optimization

The antifungal production input variables with its ranges (Table 1) were glucose concentration, temperature, and incubation time carried out using a working volume of 50 ml. The model response factor was antifungal activity (mm) (from antifungal metabolite in the fermentation broth). The response surface methodology was employed to obtain seventeen (17) independent runs (Table 2). Ten percent (5 mL) of the seed culture (10% v/v; ~ 1 × 10^6^ cells/mL), was fed into 45 mL of Luria Broth supplemented with varying glucose concentration (10–30 g/L), at varying temperature (25–45 °C), and incubation time (24–96 h) according to experimental design on Tables 1 and 2. Samples for further analysis were taken, and bacterial cells were separated using centrifugation (10 000 rpm, 20 min, 4 °C). The supernatant that contained the antifungal was then used for antifungal activity assay against *Rhizoctonia solani.* The antifungal activity data fitted the polynomial model Eq. (1) relating the input factors to the antifungal activity (response). The final polynomial equation was resolved in relation to coded factors.

1 $${\text{Y}}\, = \,\alpha_{0} \, + \,\alpha_{{1}} X_{{1}} \, + \,\alpha_{{2}} X_{{2}} \, + \,\alpha_{{3}} X_{{3}} \, + \,\alpha_{{{11}}} X_{{1}}^{{2}} \, + \,\alpha_{{{22}}} X_{{2}}^{{2}} \, + \,\alpha_{{{33}}} X^{{2}}_{{3}} \, + \,\alpha_{{{12}}} X_{{1}} X_{{2}} \, + \,\alpha_{{{13}}} X_{{1}} X_{{3}} \, + \,\alpha_{{{23}}} X_{{2}} X_{{3}}$$

**Table 1:** Modeled parameter operational range

Variables	Ranges			References
	−1	0	+ 1	
X_1_ glucose (g/L)	10	20	30	[16, 26]
X_2_ temperature (^o^C)	25	35	45	[27, 32]
X_3_ incubation time (h)	24	48	72	[27, 35]

**Table 2:** Estimated antifungal activity from model experimental runs

Run	X_1_-Glucose (g/L)	X_2_-Temperature (°C)	X_3_-Time (h)	Y_1_-Antifungal activity (mm)
1	30	35	96	52 ± 0.71
2	10	35	24	71 ± 0.18
3	20	25	24	57 ± 0.71
4	20	35	60	63 ± 0.35
5	20	35	96	52 ± 0.71
6	20	35	60	78 ± 0.18
7	20	25	96	0 ± 0.00
8	30	45	60	64 ± 0.35
9	20	45	24	61 ± 0.35
10	20	35	60	79 ± 0.18
11	10	45	60	69 ± 0.09
12	20	35	60	65 ± 0.09
13	20	35	60	52 ± 0.71
14	20	45	96	68 ± 0.09
15	30	35	24	78 ± 0.18
16	30	25	60	0 ± 0.00
17	10	25	60	63 ± 0.35

Antifungal activity = Zone of inhibition

Y represents the response factor (Antifungal activity), α_0_ is the intercept, *α*_1_*X*_1_ to α_3_*X*_3_ are the linear coefficients, *α*_11_*X*^2^_1_ to *α*_33_*X*^2^_3_ are the quadratic coefficients and *α*_12_*X*_1_*X*_2_ to *α*_23_*X*_2_*X*_3_ represents the interaction of coefficients.

The developed model was further assessed with Analysis of variance (ANOVA) and the optimal antifungal production setpoints were achieved through the resolution of the equations. These setpoints were then validated experimentally. The experimental validation was implemented using the predicted optimized input variables of 11.5 g/L (glucose concentration), 41 °C (incubation temperature) and 24 h (incubation time) to obtained high antifungal activity.

### Nanoparticle preparation and characterization

Seven nanoparticles used in this study were prepared using the co-precipitation method [38]. Nickel oxide (NiO), iron (III) oxide (Fe_3_O_4_) iron (II) oxide (Fe_2_O_3_), zinc oxide (ZnO), manganese oxide (MnO_2_), cobalt Oxide (CoO) and copper oxide (CuO) were synthesized accordingly as outlined in Sanusi et al. [38]. The obtained nanoparticles were characterized using scanning electron microscope (SEM), transmission electron microscopy (TEM) and Fourier transform infra-red spectra (FTIR).

### Nanoparticle supplementation set up

The seven nanoparticles (Fe_2_O_3_, Fe_3_O_4_, CoO, CuO ZnO, NiO and MnO_2_) were assessed for their potentials to enhance the growth and the antifungal production of *B. subtilis* BS20. Each nanoparticle type was added in an independent set up before incubation at concentrations of 0.01 and 0.05 g/L for the batch fermentation. This was undertaken using a working volume of 50 mL, with process conditions of 11.5 g/L, 41 °C and 24 h for glucose concentration, process temperature and incubation time, respectively.

### Fermentation conditions

Batch fermentations were carried out in 2L (Bio/CelliGen 115, New Brunswick, USA) and 10L (Labfors-INFORS HT, Switzerland) bioreactors under anaerobic conditions with working volumes of 1L and 10L, respectively. Luria Bertani (10 g/L tryptone, 5 g/L yeast extract, 10 g/L sodium chloride) supplemented with 11.5 g/L glucose were fed to the sterilized bioreactor and then inoculated with the seed culture (10% v/v). This was followed by fermentation process carried out at 41 °C and 120 rpm for 24 h. Samples were taken at regular intervals, the broth samples were centrifuged (5000 rpm, 20 min, 4 °C) and the supernatant was used for the antifungal activity determination.

### Process scale up

#### Scale up parameter determination

Three scale up parameters namely, constant power consumption per unit volume (*P/V*), Reynolds number (*Re*) and impeller tip speed (*V*_*tip*_) were used to determine the most suitable operational conditions for a semi-pilot scale antifungal production by *B. subtilis BS20* [21, 31, 33]*.* The details and the differences in the bioreactors’ geometry is presented in the supplementary document (Table S1). These were also used to compute (using the formulas in Table 3) the constant impeller tip speed, constant power consumption, Reynolds number (*Re*), pumping capacity (*V*_*P*_), fluid circulation time (*t*_*c*_), scale of turbulence determination (*λ*) and the shear stress ($\gamma$).

**Table 3:** Scale up parameters implemented in the study

Formula	Parameter description	Unit
Constant impeller tip speed (*V*_*tip*_) ${v}_{tip}= \pi ndi$ ${n}_{10L} = {{n}_{1L} (di}_{1L}/{di}_{10L})$	$\pi$=Pi constant $n$ = impeller speed $di$ = impeller diameter ${n}_{1L}$ = speed in 2L bioreactor ${n}_{10L}$ = speed in 10L bioreactor ${di}_{1L}$ = 2L impeller diameter ${di}_{10L}$ = 10L impeller diameter	rpm m rpm rpm m m
Constant power consumption (*P/V*) $$P/V =Constant$$ $${n}^{3}{D}^{2}=Constant$$ $${n}_{1}^{3}{D}_{1}^{2}={n}_{2}^{3}{D}_{2}^{2}$$ $$n_{2} = n_{1 } \,\left( {{{D_{1} } \mathord{\left/ {\vphantom {{D_{1} } {D_{2} }}} \right. \kern-0pt} {D_{2} }}} \right)^{{{\raise0.5ex\hbox{$\scriptstyle 2$} \kern-0.1em/\kern-0.15em \lower0.25ex\hbox{$\scriptstyle 3$}}}}$$	$$n$$= impeller speed *D* = bioreactor diameter	rpm m
Reynolds number (*Re*) $Re = \frac{{\rho ndi^{2} }}{\eta }$ ${\text{n10L}} = \text{n1L . }\text{(d}{\text{1L}}\text{/d10L}\text{)}{2}$	*ρ* = broth density *η* = broth viscosity	kg/m^3^ Pa s
Pumping capacity (*V*_*P*_) ${V}_{p}={N}_{f}n{di}^{3}$	${N}_{f}$= N_f_ is the flow number (N_f_ = 0.72 for Rushton turbine)	
Fluid circulation time (*t*_*c*_) $$t_{c} = \,{\raise0.5ex\hbox{$\scriptstyle {V_{L} }$} \kern-0.1em/\kern-0.15em \lower0.25ex\hbox{$\scriptstyle {V_{P} }$}}$$	$${V}_{L}$$= volume of the liquid phase $${V}_{P}$$ = pumping capacity	m^3^ m^3^/s
Scale of turbulence determination (*λ*) $\uplambda =\left(\frac{{V}^{3}}{\upvarepsilon }\right)$ ^1/4^	*V* = the viscosity $\upvarepsilon$ = turbulence energy per unit mass of liquid	Pa s
Shear stress ($\gamma$) $\gamma =kn$	$k$= experiential constant for Rushton impeller (*k* = 10 for Rushton turbine)	

### Analytical methods

#### Determination of biomass concentration

The cell biomass concentration (g/L) was evaluated using the bacterial cell count as a function of concentration of cells. A predetermined standard curve was prepared by determining the dry weight and corresponding cell counts at varied dilutions (1, 1/2, 1/4, 1/8 and 1/16). The biomass dry weights were obtained by centrifuging 10 mL of each dilution at 5000 rpm (Heal Force, High Speed Bench-top Centrifuge, Model: Neofuge 13, Serial No. 0610130237D, China) for 10 min. The supernatant was removed, and the biomass cell pellet was dried at 90 °C until a constant mass was obtained.

#### Antifungal activity

The samples collected for each run were centrifuged at 10000 rpm (Heal Force, High Speed Bench-top Centrifuge, Model: Neofuge 13, Serial No. 0610130237D, China) for 10 min to obtain a free-cell supernatant. Thereafter, the supernatant was added to molten potato dextrose agar (PDA) at a ratio of 1:10 (v/v) and left to solidify [22]. A mycelial plug was cut out of previously grown fungal isolate and inoculated on the amended media. The control experiment was a PDA plate inoculated with only the fungal pathogen. All plates were incubated at 30 °C for 3 days. Thereafter, the antifungal activity (zone of inhibition) was determined using Eq. 2.

2 $$ZI = \frac{GTP - GCP }{{GTP}}\, \times \,100$$

where, ZI = Zone of inhibition (%), GCP = Growth in control plate (mm) and GTP = Growth in test plate (mm).

#### *B. subtilis *BS20 growth kinetics

##### *B. subtilis* BS20 specific growth rate

The specific growth rate (µ) is the rate of change of cell population per unit of biomass concentration between the lag and the stationary stages. The specific growth rates (µ) of *B. subtilis* BS20 were calculated using Eq. 3, where *X*_2_ and *X*_1_ are biomass dry weights (g/L) at *t*_2_ and *t*_1_, respectively. This equation determines the population change rate over a specific time interval. The obtained specific growth rate values (µ) and the substrate concentration data were subsequently used to estimate the maximum specific growth rate (µ_max_).

3 $$Specfic growth rate \left( \mu \right) = \frac{{{\text{ln}}X_{2} - {\text{ln}}X_{1} }}{{t_{2} - t_{1} }}$$

##### *B. subtilis* BS20 logistic model kinetic

Integrated logistic model expresses the changes in *B. subtilis* BS20 growth as a function of cell growth rate, initial concentration, maximum cell concentration, and cultivation time. The integrated logistic equation (Eq. 4) was used in this regard. This defines the relationship of biomass dry weight (*X*), at specific times (*t*) during *B. subtilis* BS20 active growth and stationary phases to initial biomass dry weight (*X*_0_), maximum biomass dry weight (*X*_max_) and maximum specific growth rate (µ_max_) during the cultivation process.

4 $$X = \frac{{X_{0} .exp\left( {\mu_{max} .t} \right)}}{{1 - \left( { \frac{{X_{0} }}{{X_{max} }}} \right).\,\left( {1 - exp\left( {\mu_{max} .t} \right)} \right)}}$$

## Results and discussion

### Model development

High coefficient of determination (*R*^2^) of 0.86 was obtained for the designed model, indicating that the developed model could account for over 86% variability in the observed data. The suitability of the model was further assessed using analysis of variance (ANOVA) (Table 4). The model had a high *F*-value of 4.62 and *P*-value of 0.0279, which further indicate the model significance (*p* < 0.05 indicates statistically significant model) [27]. Moreover, the parameters of incubation temperature and time were found to be significant (Table 4). The noticeable influence of process temperature on antifungal production might be attributed to its impacts on *B. subtilis* BS20 metabolic activities and growth kinetics. On the other hand, high temperatures, negatively impact process performance by denaturing the cells’ enzymes, shortening the exponential growth phase thus inhibiting antifungal production. The incubation time could affect water loss through evaporation, hence, metabolic performance. Water has been identified as an important factor during bioprocessing, this is because water presence influences the dielectric properties of reacting or interacting substances. Also, water impacts  heating and mass transfer in the course of the fermentation process [3].

**Table 4:** Model analysis of variance (ANOVA)

		*R*^2^	*F*-value	*p*-value	Prob > *F*
Antifungal activity	Model		4.62	0.0279	Significant
	B: Incubation temp		13.40	0.0081	Significant
	C: Incubation time		7.14	0.0319	Significant
	Lack of fit		1.88	0.2744	Not significant
	Co-efficient of determination	0.86			

Moreover, the model polynomial equation relates the input parameters, illustrates the linear, interactive, and quadratic effect of the parameters to the antifungal activity. The resolved final polynomial mode equation in relation to coded factors is represented below (Eq. 5).

5 $$Antifungal activity \left( {mm} \right) = + 67.40 - 7.62 A + 17.13B + 12.50C + 14.50AB - 1.75AC + 14.75BC - 0.2A^{2} - 18.20B^{2} - 3.95C^{2}$$

where A = Glucose concentration (g/L), B = Incubation temperature (^o^C) and C = Incubation time (h).

### Interactive effect of input parameters

**Fig. 1:**
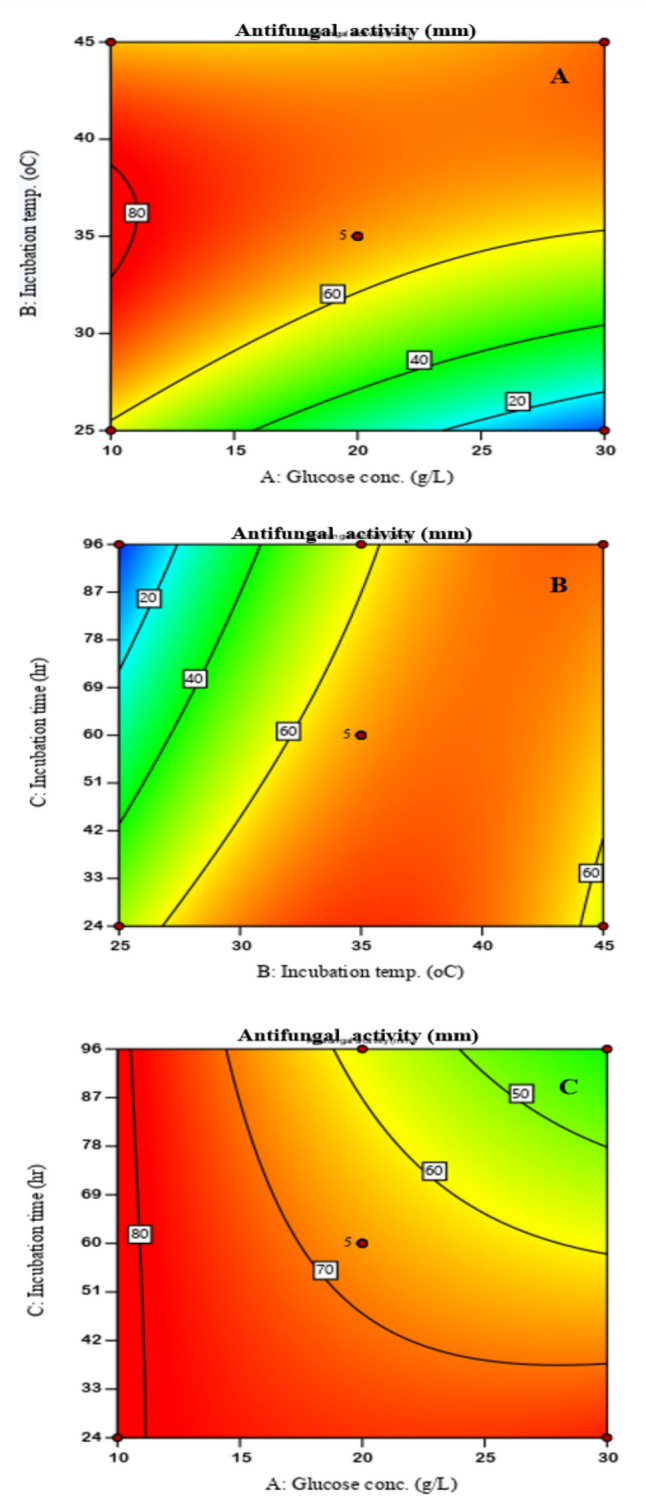
2-D plots showing the interactive effect of glucose concentration and incubation temperature (**A**), incubation temperature and incubation item (**B**), and incubation time and glucose concentration (**C**), on the anti-fungal activity potential of B. subtilis BS20 against *R. solani* phytopathogen

Response plots were obtained for the interactive effect of each pair of independent input variables on the antifungal production by *B. subtilis* BS20 (Fig. 1). Among the tested variables the incubation time and the process temperature showed significant effects on the antifungal production (antifungal activity). High antifungal activity (from antifungal produced) was obtained at initial setpoints but simultaneously increasing the glucose concentration (11–20 g/L) and incubation temperature (26–45 °C) resulted in decreased antifungal activity (Fig. 1A). Although, glucose is a major carbon source and a nutritional requirement for microbial growth and proliferation, in this study, low glucose concentration correlated with high antifungal production (antifungal activity), while high glucose concentration (> 15 g/L) resulted in repression of growth and productivity [16, 20]. Contrarily to this study. Hidmet et al. [20] observed the highest lipopeptides production by *Bacillus mojavensis* A21 using glucose concentration of 30 g/L. Also, temperature significantly affected antifungal production, as any increases in temperature resulted in decreased antifungal production. High microbial metabolism has been ascribed to optimal incubating temperature [29]. In the present study, high antifungal production was obtained at 25 °C. Ohno et al. [32], evaluated the effect of temperature on iturin and surfactin production and observed that the production of these metabolites had different optimum temperatures of 25 °C and 37 °C, respectively.

Moreover, increasing the incubation temperature (25–43 °C) while simultaneously varying the incubation time (24–60 h) resulted in antifungal production with high antifungal activity (62 mm) (Fig. 1B). Further increase in temperature (> 43 °C) resulted in slight decrease in antifungal production with high antifungal activity. Temperature is an important parameter for enzyme activity and microbial growth. At extreme temperature, enzymes are inactive, thereby limiting the formation of primary metabolites during microbial growth [28]. Optimal incubation time depends on the particular species of *Bacillus* because different species have different metabolic pathways.

Furthermore, the synergetic effect of glucose concentration and incubation time on antifungal activity showed desirable outcome (Fig. 1C). Simultaneous increase in the glucose concentration from 10 to 30 g/L and incubation time from 24 to 60 h resulted in antifungal activity from 0 to 79 mm. Any further increments in the incubation time from 60 to 96 h caused a decrease in the antifungal activity from 79 to 59 mm. Additionally, when the incubation time was maintained at its minimum value (24 h) while varying the glucose concentration (10–30 g/L), high antifungal activity of 70 mm was obtained. On the other hand, maintaining the incubation time at 96 h while glucose concentration was varied from 10 to 30 g/L resulted in a very low antifungal activity below 40 mm. Likewise, when the glucose concentration was varied from 10 to 25 g/L while instantaneously varying incubation time from 24 to 60 h occasioned high antifungal activity of 65 mm. Additional increase in the glucose concentration (> 25 g/L) showed a decrease in antifungal activity from 70 to 40 mm. The influence of incubation period in bioprocessing has also been reported by other authors [8]. Similarly, Puri et al. [35] observed that *Bacillus* sp. produced alkaline protease that showed decreased protease activity after 96 h of incubation. The result in this study shows high sensitivity of antifungal production (antifungal activity) to process input parameters, thus, it is vital to ensure that the most favorable conditions are implemented for high antifungal activity output.

### Process validation

**Fig. 2:**
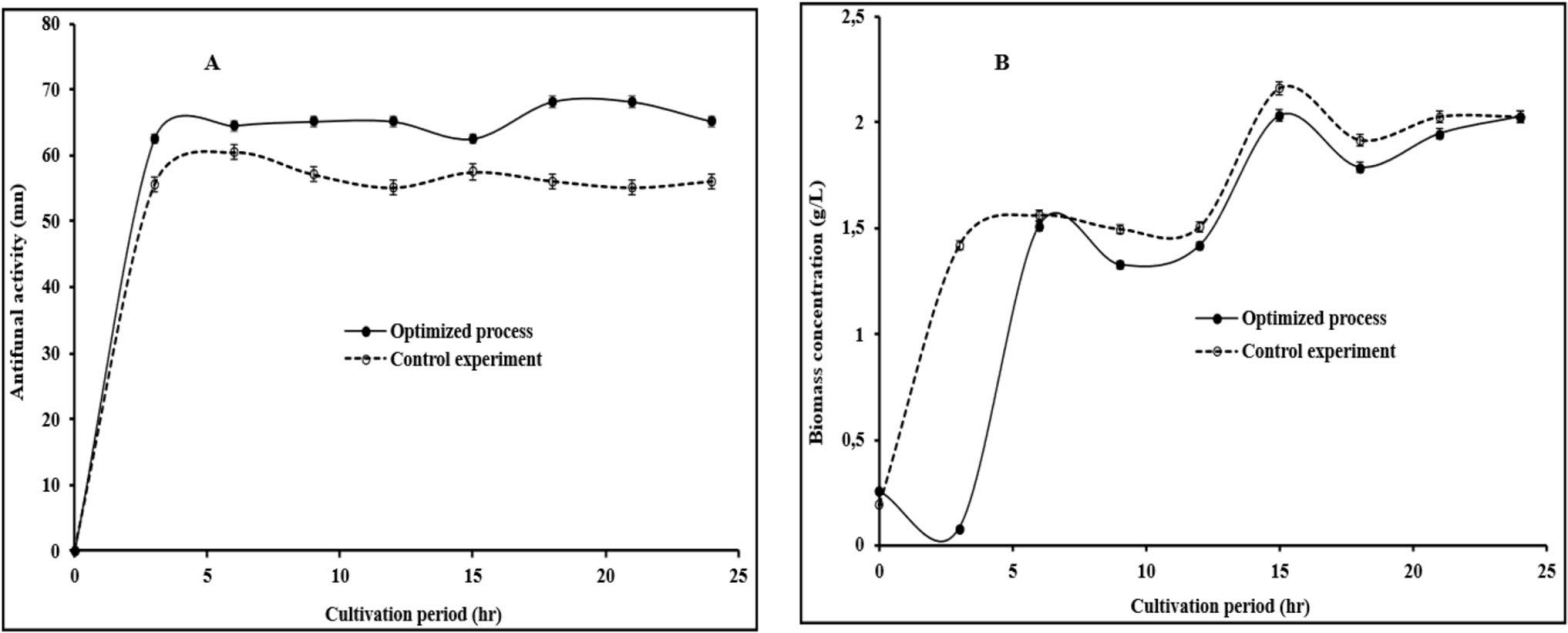
Estimated antifungal activity (**A**) and biomass concentration (**B**) of *B. subtilis* BS20 obtained under optimized conditions

The model predicted validation parameters of 11.5 g/L (glucose concentration), 41 °C (incubation temperature) and 24 h (incubation time) to obtained antifungal production with antifungal activity of 67 mm. Experimental validation carried out showed the antifungal production obtained had antifungal activity of 68 mm zone of inhibition, compared to 60 mm observed for the control experiments. Antifungal activity increased from 0 h till the 12 h (65 mm), and then maximum antifungal activity (68 mm) was obtained after the 18th hour (Fig. 2A). Maximum antifungal activity was associated with the optimized process conditions employed that provide a relatively favorable metabolic condition for antifungal production with high antifungal activity (62–68 mm). The optimized process condition showed a 12% increment in the antifungal activity over the control experiment. Similar results have been obtained by Mouafi et al. [27], the authors showed that the antifungal production obtained has high emulsification index of 71.89% under optimized conditions of 33 °C, 8, 10 h and 8.5 g/L for incubation temperature, pH, incubation period and glucose concentration, respectively. On the other hand, in the presence study, biomass concentration in the control experiment was higher compared to the optimized process as shown in Fig. 2B. Although, the biomass concentration is lower in the optimized system, antifungal production with high antifungal activity was obtained. An indication that the production of high antifungal activity metabolites is inversely proportional to biomass accumulation. Also, metabolic pathway that favors high cell proliferation do not promote high antifungal activity metabolites.

### Effect of nanoparticle supplementation

To further improve the process efficiency, nanoparticles (Fe_2_O_3_, Fe_3_O_4_, ZnO, NiO, CuO, CoO, and MnO_2_ NPs) were included in the process set up. The synthesized nanoparticles had rough spherical shape, except Co NPs which was irregular in shape (supplementary document-Fig. S1). The particle size obtained show an average diameter of 47, 31, 30, 29, 15, 12 and 8 nm was recorded for Fe_2_O_3_, Fe_3_O_4_, ZnO, NiO, CuO, CoO, and MnO_2_ NPs respectively. The variation in the average diameter may be attributed to differences in microwave irradiation treatment, precursors used and precipitation rate. Moreover, Mn–O, Co–O, Cu–O, Ag–O, Zn–O and Fe_3_O_4_ absorption band were observed at 860, 659, 845, 797, 715 and 664 cm^−1^ respectively. While NiO NPs and Fe_2_O_3_ NPs had an absorption band below 650 cm^−1^. Oxides and hydroxides of metallic nanoparticles usually give absorption peak below the wavelength of 1000 nm. This arises from inter-atomic vibrations. The other peaks observed suggested the presence of functional groups, with different stretching vibrations of –CH_3,_ -CH_2,_ = C-H, –C-H, C = O, -OH and NH groups [38]. These shape, size, elemental (metallic and oxygen) composition and surface charge of NPs are the typical features that conferred nanoparticles their catalytic properties, surface-to-volume ratio, interaction, and specificity. The chemical interactions of nanoparticles in biochemical reactions depends on these properties [38].

*B. subtilis* BS20 growth in the present of these nanoparticles is presented in the supplementary document (Table S2), with high biomass concentration but high antifungal activity was not achieved. This agrees with the result obtained in Sect. 3.3. Higher biomass accumulation in the nano-supplemented processes could be attributed to the impact of nanoparticles on *B. subtilis* BS20 (as microbes require metals like Ni, Fe, Zn, Co, Cu and Mn that are essential for microbial metabolism and growth) and the employed optimal conditions. The influencing effects of the metallic nanoparticles might as well be due to their cellular uptake in ionic form and incorporation with the metabolic intermediates and key enzyme activities. The bioavailability of metals to improve bioprocessing usually results from their dissolution to assimilable ionic form [24, 40]. For example, previous study by Liu et al. [24] reported that the release of Mn^2+^ ions from the dissolution of MnO_2_ nanoparticle might be responsible for the observed increase in bacterial activities. MnO_2_ nanoparticle like other nanoparticles ensure the availability and distribution of metallic ions in the fermentation broth. Mn^2+^ ions additives could increase the process from two aspects. First, the enhanced release of metabolite in the presence of Mn^2+^ ions additives provides a suitable metabolic environment for the bacteria. Second, Mn^2+^ ions could directly serve as an electron donor during various biochemical redox reactions. Likewise, a suitable chemical condition improves the bioavailability of metals in bioprocess and influences their functionality as well as the productivity of the system [38]. In a related study by Yang et al. [43], the authors showed that iron nanoparticle are able to increase surfactin production from 4.93 to 7.15 g/L [43]. Moreover, this high or increment in biomass accumulation was probably due to the improvement in the metabolic activities of *B. subtilis* BS20 as a result of metal-biomass interactions that depend on the chemical, biological and physical processes occurring at and near the biological interface in controlling trace metal bioavailability through shifts in the limiting bio-uptake fluxes. Also, many cellular processes are catalyzed by transported ions and mineral ions (such as obtained from nanoparticles), which improves processes like cell division, protein synthesis, and transportation of materials across the plasma membrane towards enhanced cellular activities, cell proliferation and accumulation [38]. Also, nanoparticles are known to have capacity to stimulate the formation of cytochromes and ferroxins (Fd) that are vital for cell energy metabolism towards high cellular activities. Likewise, micronutrients such as iron (Fe), nickel (Ni), cobalt (Co), zinc (Zn), silicon (Si) are essential in cellular activities due to their cellular uptake and integration with the enzyme-metabolic intermediates [38]. In addition, the effect of nanoparticles on the process can be ascribed to the oxidation–reduction potential (low value is desirable for bioprocessing), that provide a relatively good start-up environment for microbial growth and activities. Similarly, the higher biomass concentration obtained for the nano-supplemented process in this study could be of interest considering the high cell proliferation in the nano-supplemented system, the cell could be engineered for improve high antifungal activity metabolite production. This, however, may depend on the microbial strain, process conditions, biochemical properties of the metal and its interaction with other metal ions in the medium.

### Effects of scaling up on process performance

**Fig. 3:**
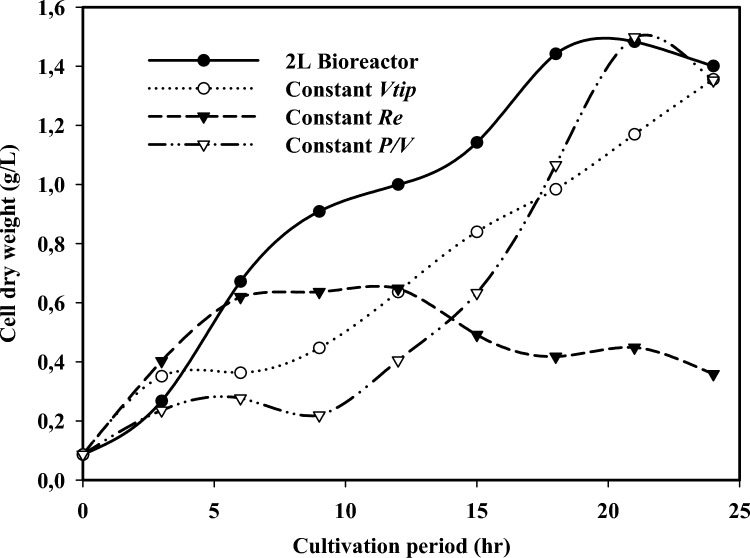
Cell dry weight in 2L bioreactor and 10L bioreactor (constant *Re*, *P/V* and *V*_*tip*_)

The differences in the rheological characteristics in the different reactors are shown in Table 5. The experimental profiles for the biomass concentration and the antifungal activity in the 1L as well as the 10L scale bioreactors are presented in Fig. 3. The maximum biomass concentration of 1.49 and 1.35 and 0.65 g/L were obtained for constant *P/V, V*_*tip*_ and *Re*, respectively. Biomass concentration obtained for constant *V*_*tip*_ and *Re* were slightly lower than that obtained in the 1L scale (1.48 g/L). This could probably be ascribed to the variation in the process environment obtained with constant *V*_*tip*_ and *Re*, which might be detrimental to cell viability and growth [13]. Furthermore, the biomass concentration (g/L) increased in the first few hours (3 h) of the process and extended till the 15th hour, this coincided with antifungal activity metabolite production during this period. Antifungal activity from *B. subtilis* BS20 metabolite (produced in 10L bioreactor using constant *V*_*tip,*_* Re* and *P/V*) effectively inhibits fungal growth (Table 6). Antifungal activity from impeller tip speed bioreactor had the highest antifungal activity of 65 mm [19]. This was 1.14, 1.18, and 1.38-fold higher than the antifungal activity obtained with antifungal metabolite from 1L, power consumption (10L) and the Reynolds number (10L) bioreactors respectively. This result suggests constant *V*_*tip*_ process conditions support metabolic activities that favor the production of high efficacious antifungal activity metabolites.

**Table 5:** Bioreactor geometry employed in the scale up processes

1 L control bioreactor		10 L bioreactor		
Parameters		Constant ʋ_tip_	Constant P/V	Constant $Re$
n (rpm/rps)	120/2	93/1.55	88/1.47	71/1.18
ʋ_tip_ (m/s)	0.34	0.34	0.32	0.26
Re	4.5 ˟ 10^–4^	5.9 ˟ 10^–4^	5.6 ˟ 10^–4^	4.5 ˟ 10^–4^
P (W)	0.0156	0.32	0.0156	0.32
P/V_L_ (W/m^3^)	15.62	160	7.8	160
*V*_*P*_ (m^3^/s)	2.3 ˟ 10^–4^	3.4 ˟ 10^–4^	3.6 ˟ 10^–4^	2.9 ˟ 10^–4^
*t*_c_ (s)	4.4	5.2	5.5	6.9
λ (m)	17.5	15.6	16.3	19.2
γ (1/s)	20	15.5	14.7	11.8

**Table 6:** Fermentation performance for the scale up studies

1 L control bioreactor		10 L bioreactor		
Parameters		Constant *V*_*tip*_	Constant *P/V*	Constant *Re*
Biomass concentration (g/L)	1.5	3.097	4.129	0.477
Antifungal activity (mm)	57	65	55	47

Meanwhile, the antifungal activity indicated by zone of inhibition was lower in the 10L scale for *P/V* (55 mm) and *Re* (47 mm) compared to the 1L scale (57 mm) (Table 6). The variation in antifungal activity response under various fermentation scales might be ascribed to differences in rheological characteristics and the bioreactor geometry in the different reactors. The rheological characteristics of the nutrient broth changes during the process as biomass and products accumulate (Perez et al., 2018). Thus, the velocity and turbulence of fluid leaving the stirrer must be adequate to carry material into the most remote parts of the bioreactor to ensure and maintain effective mixing regime. The performance of a reactor is influenced, to a very significant extent, by mixing effects on metabolic processes and productivity. Additionally, the liquid volume that was dismissed from the stirrer per unit time (*V*_*p*_) and the circulation time (*t*_*c*_), is another important quantitative mixing characteristics to obtain good mixing regime. This must have contributed to the process performance in the bioreactors.

#### Scaling up based on constant V_tip_

The process outcome obtained based on constant *V*_*tip*_ experiment had the highest antifungal activity of 65 mm zone of inhibition (Table 6). This was also higher (12%, 15% and 28%) when compared to the result obtained in the 1L scale bioreactor, the constant *P/V* (10L) and *Re* (10L) experiments, respectively. These results (lower antifungal activities using constant P/V and Re) might be ascribed to the lower mixing rate employed due to constant *P/V* and *Re* at 10 L scale (Table 5). Moreover, scale up based on maintaining *P/V* resulted in lower shear stress of 11.8 compared to constant impeller tip speed (15.5). This increase in the shear stress may result to probable cell damage as well as affect the cell metabolic physiology and consequently, decrease in cellular productivity of desired interest. Although, excess shear stress could lead to the loss of cell viability and cell disruption, a certain degree of shear rate is necessary to achieve appropriate transfer of materials and energy within the bioreactor. Impeller tip speed influences impeller shear, which is proportional to the product of impeller tip speed and impeller diameter for suitable turbulent flow conditions [14, 25].

#### Scaling up based on constant power consumption P/V

Based on constant *P/V*, antifungal activity of 55 mm zone of inhibition was obtained. Antifungal activity obtained with the 1L bioreactor was 1.07-fold higher in comparison with the 10L scale *P/V* experiment (Table 6). These can be elucidated from the mixing viewpoint of the fluid homogenization level [9, 11]. Regardless of the flow regime achieved in the 10L scale set up, the flow will remain laminar at micromixing scale, due to its larger surface area and double impeller system employed [41]. Hence, resulting in a mixing regime probably lower than the optimal process parameters. Other reasons for lower antifungal activity in the constant *P/V* experiments could be ascribed to the different geometrical, rheological, and hydrodynamic parameters implemented. Moreover, using the constant power consumption criterion, the size of eddies was computed to be 16.3 m which was considerably larger than an average *B. subtilis* BS20 cell size. When the Kolmogorov eddy size equals the cell diameter, the flow lines pattern could shear growing cells [13]. On the other hand, smaller eddies, facilitates rapid transfer of material, which is proportionate to the power input. The greater the power input to the fluid, the smaller are the eddies, the better the mixing regime and consequently, the better the system productivity [36]. Similarly, the present outcome could be related to the turbulent flow, since the power number for the current scale up was 5.20 at fully turbulent flow and 10 for the characteristic experiential constant (*k*) for a standard Rushton turbine impeller [13].

#### Scaling up based on Reynolds number

Constant Reynolds number showed antifungal activity of 47 mm zone of inhibition, this was lower compared to the 1L bioreactor, *P/V* and* V*_*tip*_ experiments (Table 6). Scaling up with the constant Reynolds number criterion, has very low values for impeller agitation speed in the 10L scale bioreactor. This low impeller speed might provoke an inappropriate mixing, resulting to negative effect on *B. subtilis* BS20 metabolic activities, proliferation and consequently lower values for biomass concentration and antifungal activity obtained at 10L scale. This agrees with literature that Reynolds number as scaling up criterion usually result in adverse impact on the process. This is because the degree of agitation decreases very rapidly with the increase of the production scale using Reynolds number as scaling up criterion [34]. Unfortunately, very low impeller speed might physically be impracticable to maintain desirable process conditions at large scale. The reason being that physical processes are dimensionally related while metabolic processes are indirectly scale dependent. This could lead to improper mixing regime affecting the growing cell’s physiology, metabolic activities, and productivity as it was obtained with constant Reynolds number in the present study. In a related study, Obonna et al. [31], also, reported that implementing the same or lower mixing speed used in a 1L bioreactor was not appropriate for the 8L bioreactor. Hence, the cells and the substrate were not homogeneously distributed in the large-scale bioreactor. Mixing rate could influence the mass transfer and temperature gradient homogeneity adversely for high viscous fermentation broth [13]. Also, studies have shown that when a scaling up approach resulted in an increased Reynolds number a low *P/V* value is obtained, which is not sufficient for efficient mixing, leading to probable death regions, hence, productivity rate is adversely affected [13].

## Conclusion

In this study, optimization, and nano supplementation for improved antifungal activity metabolite production were achieved. Model developed elucidates functional relationships with maximum antifungal activity of 68 mm obtained with 11.5 g/L glucose concentration at 41 °C incubation temperature after 24 h. Moreover, while inclusion of nanoparticles (NPs) significantly improved biomass concentration lower antifungal activity was observed compared to the control experiment. This could be ascribed to the potential of nanoparticles to enhance transportation of ions and mineral elements within the cell, which help to improve cellular activities such as cell division, protein synthesis, and transportation of materials across the plasma membrane which in turn enhance cell proliferation and accumulation but not antifungal metabolite production. Also, this study has provided a simple but coherent rheological model for translating an optimized laboratory scale antifungal activity metabolite production to a pilot scale successfully. Constant *V*_*tip*_ was a better approach in scaling up the production of antifungal by *B. subtilis* BS20 due to the suitable mixing regime, pumping capacity (3.4 × 10^–4^ m^3^/s), circulation time (5.2 s) and turbulence (15.6 m). Maintaining a constant *V*_*tip*_ upon scaling up from 1 to 10L scale, higher antifungal activity (65 mm) was achieved. The findings have provided valuable insight for high antifungal activity metabolite production using *B. subtilis* BS20 with potential for industrial scale production.

## Supplementary Information

Below is the link to the electronic supplementary material.

Supplementary file1 (DOCX 777 KB)

## Data Availability

No datasets were generated or analyzed during the current study.
