# Supplementary material for: Process development for antifungal production by Bacillus subtilis BS20: nanoparticle supplementation, process optimization and preliminary scale-up studies
Source: Bioprocess Biosyst Eng. 2025 Jul 26;48(10):1707–18. doi: 10.1007/s00449-025-03205-6 (PMC12460514; doi:10.1007/s00449-025-03205-6)
Supplement: Supplementary file 1 — Supplementary file1 (DOCX 777 KB) [file 449_2025_3205_MOESM1_ESM.docx]

**Supplementary material**


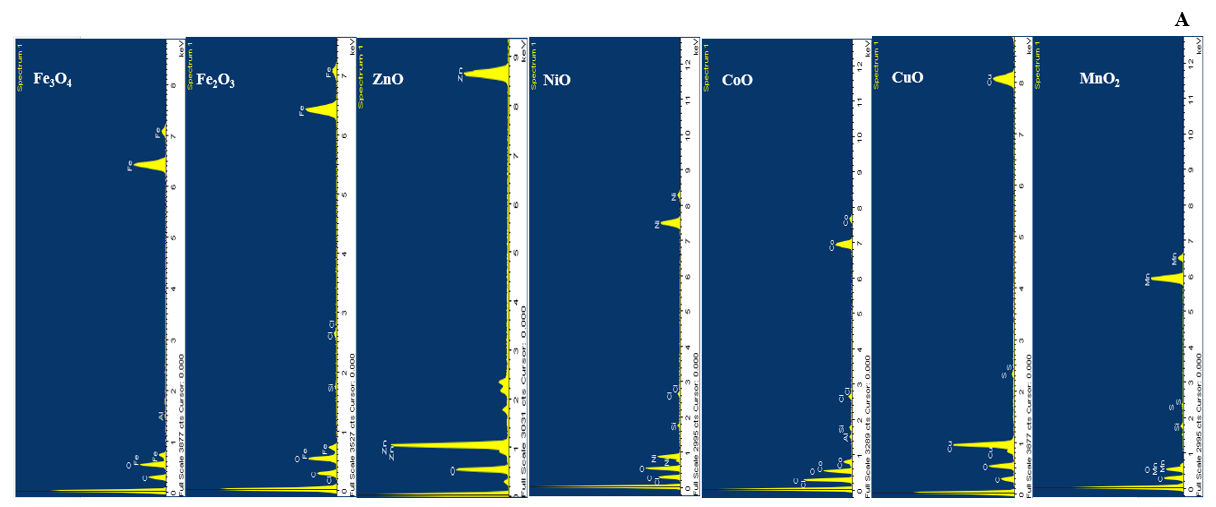


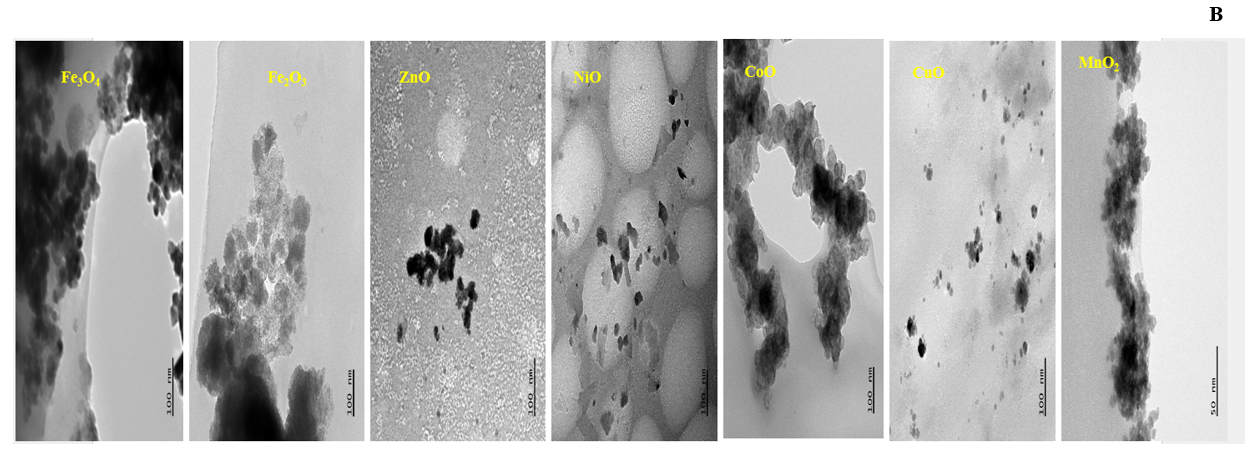


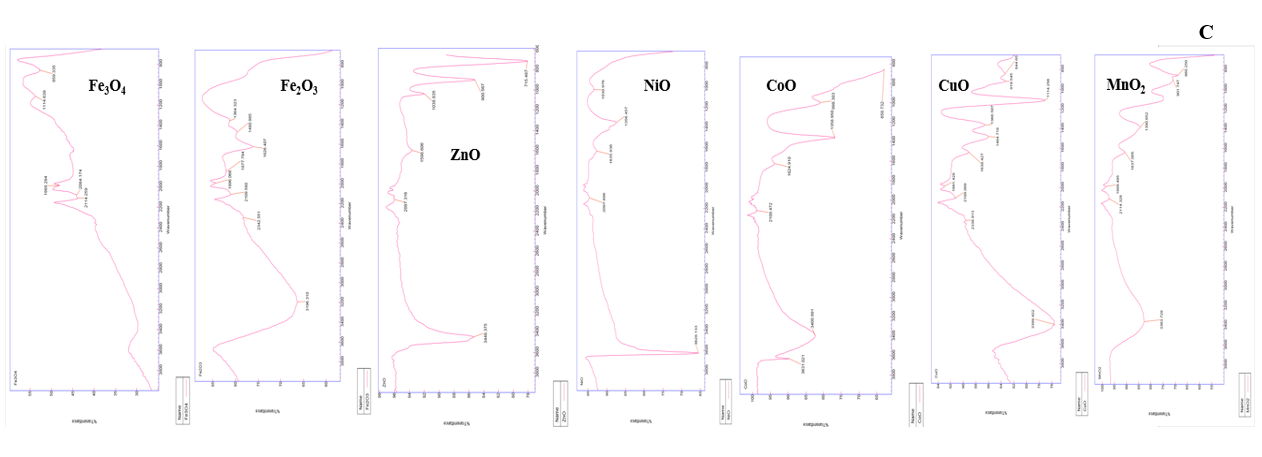


**Fig. S1: SEM-EDX (A), TEM (B) and FTIR (C) images of the various synthesized nanoparticles**

**Table S1: Bioreactor geometry employed in the scale up processes**

| Parameters | 1 L scale | 10 L scale |
| --- | --- | --- |
| Total bioreactor volume (m^3^) | 0.002 | 0.010 |
| Working volume (m^3^) | 0.001 | 0.005 |
| Bioreactor height [h] (m) | 0.237 | 0.427 |
| Bioreactor diameter [D] (m) | 0.125 | 0.200 |
| Static height of broth [H] (m) | 0.084 | 0.162 |
| Number of impellers (N) | 1 | 2 |
| Impeller diameter [di] (m) | 0.054 | 0.070 |
| Impeller thickness (m) | 0.001 | 0.002 |
| Power number (N_p_) | 5.20 | 10.40 |
| Broth density [ρ] (kg/m^3^) | 1013 | 1013 |
| Broth viscosity [ƞ] (Pa s) | 9.173 ˟ 10^-5^ | 9.173 ˟ 10^-5^ |
| Impeller type | Rushton turbine | Rushton turbine |

**Table S2: Effect of nanoparticles on antifungal activity and biomass concentration**

| NPs concentration (0.01g/L) | AA (mm) | Biomass concentration (g/L) | NPs concentration (0.05 g/L) | AA (mm) | Biomass concentration (g/L) |
| --- | --- | --- | --- | --- | --- |
| Fe_2_O_3_ | 59 | 1.24 | Fe_2_O_3_ | 27 | 2.59 |
| Fe_3_O_4_ | 58 | 1.87 | Fe_3_O_4_ | 44 | 2.80 |
| ZnO | 51 | 3.81 | ZnO | 33 | 10.28 |
| MnO_2_ | 61 | 2.94 | MnO_2_ | 51 | 3.41 |
| CuO | 0 | 2.95 | CuO | 0 | 0.05 |
| CoO | 61 | 2.89 | CoO | 0 | 0.29 |
| NiO | 65 | 5.11 | NiO | 63 | 3.43 |
| Control | 68 |  | Control | 68 |  |

**AA=Antifungal activity**
